# Chemical Pneumonitis Caused by the Inhalation of Zinc Oxide Fumes in an Arc Welder

**DOI:** 10.3390/ijerph19137954

**Published:** 2022-06-29

**Authors:** Eun-Woo Cha, Doosoo Jeon, Dongmug Kang, Young-Ki Kim, Se-Yeong Kim

**Affiliations:** 1 Department of Occupational and Environmental Medicine, Pusan National University Yangsan Hospital, Yangsan 50612, Korea; rhodus19@pnuyh.co.kr (E.-W.C.); kangdm@pusan.ac.kr (D.K.); mungis@pusan.ac.kr (Y.-K.K.); 2 Department of Internal Medicine, Pusan National University Yangsan Hospital, Yangsan 50612, Korea; doosooj@pusan.ac.kr; 3 Department of Preventive and Occupational & Environmental Medicine, Medical College, Pusan National University, Yangsan 50612, Korea

**Keywords:** chemical pneumonitis, zinc oxide fumes, welding

## Abstract

Acute respiratory illness caused by exposure to welding-associated zinc oxide fumes is known as metal fume fever (MFF). MFF is generally characterized as a self-limiting disease. Few studies have reported chemical pneumonitis associated with zinc fume inhalation. We report a case study involving severe episodes of MFF accompanied by chemical pneumonitis due to the inhalation of zinc oxide fumes while operating an arc welder. A 54-year-old man developed flu-like symptoms after arc welding galvanized steel in a poorly ventilated area. Despite intravenous antibiotics therapy, his clinical course worsened, and his urine zinc concentration was remarkably elevated (3579 μg/24 h; reference range, 0–616 μg/24 h). A chest computed tomography revealed extensive consolidation, ground-glass opacity in the lungs, and right pleural effusion. After corticosteroid treatment, the patient’s symptoms and radiologic findings significantly improved. It should be noted that the inhalation of zinc oxide fumes can occasionally induce acute lung injury via inflammatory responses and oxidative stress.

## 1. Introduction

Welding is a process wherein metals are made to coalesce by heating or applying pressure with or without the use of a filler metal [1]. Among the different types of welding, electric arc welding is the most common. During electric arc welding, metal vapors form at extreme temperatures (>4000 °C) and subsequently cool and condense into fumes. Metal fumes typically comprise fine and ultrafine particles with aerodynamic diameters of 0.01–1 µm, which can accumulate in terminal bronchioles or alveoli and have deleterious effects on the respiratory system [2,3].

The health effects caused by the inhalation of these metal fumes have been extensively studied. Several studies have investigated the effects on the respiratory system of workers exposed to metal fumes. The most frequently reported acute respiratory illness in welders is metal fume fever (MFF) [4,5]. In addition to the typically benign and self-limited course of MFF, some severe metal fume inhalation-related lung parenchymal disorders have been reported. These cases prominently reported dyspnea, along with extensive chemical pneumonitis, acute non-cardiogenic pulmonary edema, or reactive airway dysfunction syndrome [6,7,8,9]. Generally, chemical pneumonitis caused by exposure to high concentrations of fumes from metals, such as cadmium, chromium, nickel, mercury, cobalt, and beryllium, have been well studied [6,10]. However, chemical pneumonitis caused by the inhalation of zinc fumes (zinc oxide) has rarely been reported. In the literature, two cases of chemical pneumonitis among workers who were involved in the welding or cutting of galvanized steel are studied. These workers presented with unusual diffuse alveolar damage and aseptic meningitis with pericarditis, pleuritis, and pneumonitis [11,12]. Zinc is an essential nutrient in humans and animals and is required for the functioning of a large number of metalloenzymes. However, high concentrations of zinc in the body may have deleterious health effects. When workers are exposed to high concentrations of zinc fumes, it can be deposited in the lungs. A number of acute exposure assessments of zinc fumes associated with the development of febrile respiratory diseases have not been consistent, though are generally noted at airborne zinc oxide levels of 77–600 mg zinc/m^3^ [13].

Herein, we report a patient with chemical pneumonitis who presented with high urinary zinc levels, presumably caused by welding-associated exposure to zinc oxide fumes.

## 2. Case Report

A 54-year-old man with a 30-year history of occupational welding visited the emergency room with a 5-day history of dry cough, headache, dyspnea, low-grade fever, and vomiting. The patient had been performing the arc welding of galvanized steel plumbing in an underground ditch 5 days prior. Although he had been wearing a personal protective mask, the work environment had poor ventilation as it was underground. He developed fever, chills, headache, and vomiting after returning home from work on that day, followed by a worsening headache and nausea. More than a decade ago, he had been exposed to welding fumes and had developed intermittent headaches and nausea for 3 days before spontaneous improvement. However, these symptoms were persistent and did not spontaneously improve this time. He consumed alcohol occasionally but not illicit drugs; he had no smoking history and no known allergies to food or medications.

On physical examination, he had a body temperature of 37.6 °C, a blood pressure level of 100/60 mmHg, a pulse rate of 84 beats/minute, and a respiratory rate of 20 breaths/minute. No neurological symptoms, such as neck stiffness, were reported. Laboratory tests revealed increased serum high-sensitivity C-reactive protein (hs-CRP) levels, and his liver function tests gave abnormal results, with raised aspartate aminotransferase (AST) and alanine aminotransferase (ALT) levels. Arterial blood gas analyses revealed a pH of 7.49, PaCO_2_ of 33 mmHg, PaO_2_ of 94 mmHg, HCO_3_^−^ of 25.1 mEq/L, and SpO_2_ of 97.9%. No abnormal findings were found on an electrocardiogram. A chest computed tomography (CT) revealed dependent atelectasis of the basal aspect of the right lower lobe.

**Figure 1:**
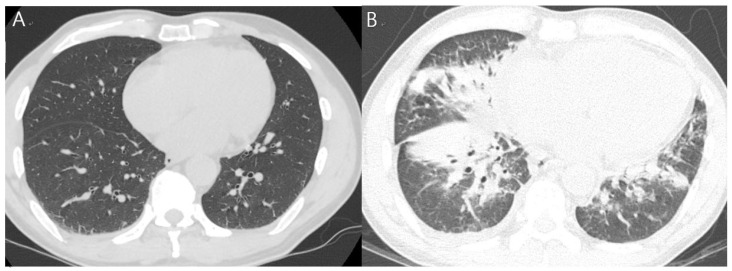
Chest computed tomography (CT). (**A**) Chest CT on admission demonstrating probably dependent atelectasis of the basal aspect of the right lower lobe and (**B**) on day 6 after admission indicating new-onset extensive consolidation and ground-glass opacity of the right middle lobe, lingular segment, and both lower lobes with increased right pleural effusion.

The patient was admitted to the department of respiratory medicine of our hospital and was started on empirical antibiotics (ciprofloxacin and doxycycline). The next day, tests revealed increased levels of WBC (11,540 cells/μL; segmented neutrophils, 88.8%) and hsCRP (24.58 mg/dL). Fever and headache persisted on day 4 of admission, with a further increase in hsCRP levels (34.21 mg/dL). Considering the worsening of the symptoms, the antibiotic therapy was changed to include levofloxacin and piperacillin–tazobactam. A follow-up chest CT on day 6 of admission revealed new findings of extensive lung consolidation and ground-glass opacities in the right lower lobe, with pleural effusion increasing on the right side (Table 1, Figure 1).

No bacterial growth was noted in the cultures of peripheral blood or sputum. The tests for serum *Myoplasma pneumoniae*, *Chlamydia pneumoniae*, urinary *Legionella* antigens, and *Streptococcus pneumonia* antigens were all negative. Respiratory virus panel tests did not detect adenovirus, coronavirus, human metapneumovirus, human rhinovirus/enterovirus, influenza, parainfluenza, respiratory syncytial virus, *Bordetella pertussis*, *C. pneumoniae*, or *M. pneumonia*. Despite intravenous antibiotic therapy, the patient’s symptoms worsened, and his WBC and hsCRP levels continued to be high. The patient was referred to the department of occupational and environmental medicine. More details about his history in terms of antigen inhalation were obtained through a job-exposure matrix. Biological exposure indices were examined, as it was suspected that the patient was significantly exposed to welding-associated metal fumes, including zinc oxide fumes generated during the welding of galvanized steel sheets. The concentrations of zinc, cadmium, manganese, copper, and nickel in the patient’s blood and urine were additionally measured on day 5 of admission. Serum cadmium, copper, and manganese levels were slightly higher than the normal levels. The zinc concentration in the serum was in the normal range but that in urine was significantly elevated to more than five times (3579.04 μg/24 h) the reference value (0–616 μg/24 h) (Table 2).

**Figure 2:**
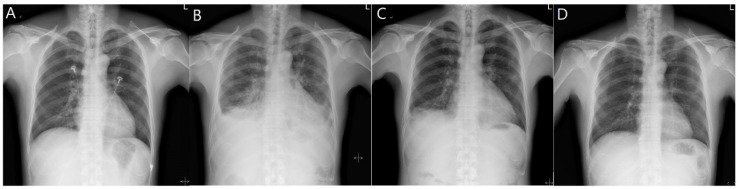
Changes in plain chest radiography (PA) with the clinical course. (**A**) No active disease at the time of admission. (**B**) Deterioration during hospitalization (6 days after admission); pneumonia or pulmonary edema; bilateral pleural effusion or thickening. (**C**) Improvement in pulmonary edema 13 days after admission. (**D**) No active disease at the time of outpatient follow-up (7 days after discharge).

Although we were unable to obtain the gas and dust samples he may have inhaled by measuring the working environment, or determine zinc particles in the lungs (bronchoalveolar lavage fluid or tissue), we diagnosed acute lung injury induced by the inhalation of zinc or zinc oxide fumes based on the obtained findings and the patient’s clinical course. A high-dose steroid therapy (methylprednisolone 31.25 mg/day) was started on day 7 of admission. The patient’s headaches and respiratory symptoms were relieved from the third day after steroid administration (day 10 of admission). He was discharged on day 14 of admission based on the chest X-ray imaging results, and his subjective symptoms remarkably improved as the levels of serum markers decreased. However, pulmonary function testing on the day of discharge revealed a persistent restrictive ventilation defect (FVC, 60%; FEV1, 61%; FEV1/FVC, 80%). During an outpatient visit 7 days after discharge, the patient had normalized serum inflammatory levels and significantly improved pulmonary edema (Figure 2).

## 3. Discussion

We report a patient with chemical pneumonitis presumably caused by the inhalation of zinc oxide fumes generated during an arc welding. This case was not easily distinguishable from infectious pneumonia. Approximately 2 h after inhaling highly concentrated welding fumes in a poorly ventilated work environment, the patient developed flu-like symptoms and reported experiencing a metallic taste. However, an extensive bilateral consolidation, ground-glass opacity, and non-cardiogenic effusion in both lungs progressed rapidly, along with fever and respiratory distress. His subjective symptoms and chest radiologic findings did not improve after antibiotic treatments, but remarkably improved soon after methylprednisolone treatment, although restrictive ventilation defects were maintained.

Chemical pneumonitis induced by exposure to metal fumes among occupational welders has previously been reported [8,11,12,14,15]. In the absence of personal protective equipment in the working environment, a worker was exposed to nickel fumes from metal arc processing and developed chemical pneumonitis and acute lung injury. A chest CT revealed interlobular septal thickening and bilateral non-segmental patchy ground-glass opacities throughout the lungs except the sub-pleural zone. Similar to our case, the patient’s symptoms continued to worsen after exposure and improved markedly after corticosteroid treatment (50 mg/day) [8]. In another case, a man used a butane torch to cut alloys comprising 10% cadmium for 60–70 min. Four hours after finishing the work, he experienced progressive dyspnea and developed fatal chemical pneumonitis. He was initially diagnosed with pneumonia, which progressed to acute respiratory insufficiency. However, the diagnosis was changed to chemical pneumonitis due to cadmium fumes based on the negative microbiological results, his occupational history, and the high concentration of cadmium in his urine [14]. Few studies have reported acute lung injury or chemical pneumonitis caused by the inhalation of zinc fumes. In a military report, 20 soldiers exposed to zinc chloride smoke presented with bilateral ground-glass opacities as the most extensive abnormality on an initial chest CT, and generally had a restrictive type of pulmonary functional impairment [15]. In another case report similar to ours, two male welders (aged 29 and 51 years) were exposed to zinc oxide fumes while using an acetylene torch to dismantle galvanized steel in a poorly ventilated area. Their treatment involved an unusually aggressive clinical course for diffuse alveolar damage in both lungs [11]. In another case, a 25-year-old male welder who used to cut galvanized steel presented with aseptic meningitis, pericarditis, pleuritic pain, and pneumonitis. He inhaled substantial metal fumes without wearing a respirator in the working environment. Consequently, his respiratory symptoms progressed to diffuse myalgia, headache, neck stiffness, and dyspnea. Symptoms and oxygenation worsened despite intravenous antibiotic therapy. Based on the patient’s clinical presentation, it was postulated that the inhalation of metal fumes, such as zinc oxide fumes, can induce a cytokine-mediated systemic inflammatory response [12].

The most widely posited causes of lung injury due to zinc oxide nanoparticles are inflammatory responses and oxidative stress. Zinc oxide-induced inflammatory responses are associated with increased levels of cytokines, including tumor necrosis factor-α (TNF-α), interleukin (IL)-6, and IL-8, thereby promoting the recruitment of inflammatory cells. Inflammatory cytokines may be produced by the activation of neutrophils, macrophages, or pulmonary epithelial cells in response to direct contact with deposited zinc oxide nanoparticles. The toxicity of zinc oxide nanoparticles may also be due to increased oxidative stress, which causes cell damage through free radicals and peroxides. Recently, increasing evidence has indicated that soluble zinc ions released from zinc oxide nanoparticles directly induce toxicity [16,17,18,19]. A bronchoscopy can be performed in cases of unexplained pneumonia. However, a bronchoscopy was not performed for our patient. A previous experimental study included 15 healthy volunteers who inhaled purified zinc oxide fumes with a mean concentration of 33 mg/m^3^ for 10, 15, and 30 min. Subsequently, the bronchoalveolar lavage fluid samples were tested for proinflammatory cells and cytokines. The results revealed a significant increase in the levels of TNF and IL-8, indicating an early inflammatory response. A zinc oxide concentration of >30 mg/m^3^ may cause inflammation in the lungs [17]. Although these variables were not actually measured in our patient, he may have been exposed to significant amounts of zinc oxide fumes during the welding of galvanized steel. A previous review reported that exposures to low concentrations of zinc oxide fumes (8–12 mg zinc/m^3^ for up to 3 h; 14 mg/m^3^ for 8 h) did not induce symptoms of MFF [20].

Diagnosing chemical pneumonitis due to the inhalation of metal fumes is difficult because the clinical symptoms may also be caused by bacterial pneumonia, influenza, or viral upper respiratory illnesses. Based on previous cases, a temporal relationship between exposure to metal fumes and compatible signs or symptoms; negative microbiological findings; analysis of the biological exposure index of serum or urine; and radiological findings such as diffuse consolidation or ground-glass opacities in both lungs are helpful in the diagnosis of these diseases [8,11,12,14,15]. Radiologically, infectious pneumonia is most commonly reported in the form of bronchopneumonia. In contrast, patients with pneumonitis generally have diffuse ground-glass opacities in both lungs, multifocal patchy consolidation, and segmental or lobar consolidation on a chest CT [21,22]. Our patient was also initially diagnosed with infectious pneumonia. However, the patient showed the aggressive clinical course despite antibiotics treatment, negative microbiological findings, the temporal relationship of exposure with symptoms, detailed occupational and environmental exposure assessments through interviews, radiological findings, and the detection of high urinary zinc concentrations. Thus, the diagnosis was changed to chemical pneumonitis due to the inhalation of metal fumes during welding. Through the biological exposure monitoring of metal fumes, we found that the serum cadmium concentration was slightly elevated (although this level could be considered in the normal range) and urinary zinc concentrations were significantly increased (3579 μg/24 h; reference range, 0–616 μg/24 h) on day 5 of admission. We suggest considering the inhalation of zinc oxide fumes as the cause of chemical pneumonitis, although complex exposure to other gases or metals, such as cadmium, produced by welding also needs to be considered.

The treatment of chemical pneumonitis is supportive care. Although its clinical efficacy is yet to be fully established, some reports have advocated the use of corticosteroids. In a case similar to ours, wherein the patient had chemical pneumonitis caused by white phosphorus, treatment with corticosteroids led to remarkable improvements in imaging findings and symptomatic relief [8,11,23]. Marked improvements in imaging findings and clinical symptoms were also observed after methylprednisolone treatment in our patient. Clinical responses to steroid therapy may support the role of cytokines and free oxygen radical injury in the pathogenesis of acute lung injury caused by the inhalation of zinc oxide.

## 4. Conclusions

A 54-year-old male arc welder developed acute respiratory illness 2 h after welding galvanized steel. Bilateral extensive consolidation, ground-glass opacity, and pleural effusion were observed during radiological examination, which were consistent with the characteristics of chemical pneumonitis. To the best of our knowledge, the cases of chemical pneumonitis associated with exposure to zinc oxide fume are few. It should be noted that the inhalation of zinc oxide fumes can occasionally induce acute lung injury via inflammatory responses and oxidative stress. Additionally, complex exposure to various welding fumes should be considered. Because it is difficult to distinguish from infectious pneumonia, clinicians should be aware of acute lung injury induced by metal fumes such as ours. The present case highlights the occupational risk of exposure to metal fumes (including zinc oxide) among welders. Moreover, preventive strategies should be used in welding environments to ensure workplace safety, including education, proper ventilation, and the provision of personal protective equipment.

## Figures and Tables

**Table 1:** Laboratory findings from the time of admission to discharge and at outpatient follow-up.

	Admission (#1)	#2	#4	#7	#10	#13	Outpatient (#20)	Reference Level	Unit
WBC	6840	11,540 *	12,950 *	16,890 *	12,210 *	11.700 *	9.840	4000–11,000	10^3^/μL
Hb	13.7	12.6	10.6	10.3	10.2	10.9	12.8	13.5–17.5	g/dL
Platelet	179	182	257	426 *	507 *	552 *	504 *	140–400	10^3^/μL
BUN	19.2	14.2	13.6	10	11.8	12.3	21.5	6.6–23.6	mg/dL
Creatinine	0.88	0.81	0.76	0.6	0.65	0.74	0.75	0.67–1.17	mg/dL
eGFR(MDRD)	>60	>60	>60	>60	>60	>60	>60	>60	mL/min/1.73 m^2^
AST	126 *	94 *	43	20	19	22	39	0–50	IU/L
ALT	128 *	136 *	105 *	63 *	40	52	75 *	0–50	IU/L
hsCRP	14.73 *	24.58 *	34.21 *	28.96 *	9.52 *	10.38 *	0.67	0–0.5	mg/dL

* Above the reference level. The patient was started on steroid treatment (methylprednisolone 31.25 mg/day) on day 7 of admission, and a significant symptom improvement was noted. The patient was discharged on day 14 of admission.

**Table 2:** Serum and urine metal concentrations (sampled on day 5 of admission).

Metal	Concentration	Reference Level	Unit
Zinc (blood)	67.55	70.00–120.00	μg/dL
Zinc (urine)	3579.04 *	0–616.0	μg/24 h
Cadmium (blood)	1.01 *	<0.90	μg/L
Manganese (blood)	8.1 *	<8.0	μg/L
Copper (blood)	155.76 *	64.0–134.0	μg/dL
Nickel (blood)	0.7	<2.0	μg/dL

* Above the reference level; urinary zinc level of patient was remarkably elevated.

## Data Availability

Not applicable.
